# Electrochemical Behavior and Highly Sensitive Voltammetric Determination of Doxepin in Pharmaceutical Preparations and Blood Serum Using Carbon Ionic Liquid Electrode

**Published:** 2019

**Authors:** Fatemeh Farjami, Farshid Fasihi, Forough Alimohammadi, Seyed Esmaeil Moradi

**Affiliations:** a *Pharmaceutical Sciences Research Centre, Shiraz University of Medical Sciences, Shiraz, Iran.*; b *Department of Chemistry, Marvdasht Branch, Islamic Azad University, Marvdasht, Iran. *; c *Young Researchers and Elite Club, Marvdasht Branch, Islamic Azad University, Marvdasht, Iran.*

**Keywords:** Carbon ionic liquid electrode, Doxepin, Tricyclic antidepressants, Voltammetry

## Abstract

In this manuscript, the electrocatalytic oxidation of doxepin (DOX) was studied at a carbon ionic liquid electrode, fabricated using graphite, and the ionic liquid 1-octylpyridinium hexaflourophosphate (OPFP). The surface of the proposed electrode was characterized by scanning electron microscopy. Differential pulse voltammetry was applied as an analytical technique for quantification of sub-micromolar concentration of doxepin. Various parameters were optimized for practical application. Under the optimal conditions, the proposed electrode exhibited interesting sensitivity toward determination of doxepin compared to the other conventional electrodes and the anodic peak current versus doxepin concentration was linear in the ranges of 0.05-24 µM. The detection limit of 21 nM was achieved. Cyclic voltammetry (CV) was also applied to acquire information about the reaction mechanism and calculating the kinetic parameters. The electroxidation process was irreversible and revealed adsorption controlled behavior. The method was successfully applied for determination of doxepin content in pharmaceuticals and blood serum samples.

## Introduction

Tricyclic antidepressants (TCAs) are one of the primary classes of antidepressants which are generally used for treatment of psychiatric disorders (1). TCAs are widely metabolized by demethylation and oxidation (2). However, they comprise severe adverse side effects and toxicity at high concentrations (3). The therapeutic concentration range for most TCAs is approximately between 280-890 nM, whereas toxic effects can occur when plasma concentrations exceed 1.4 µM (4). Doxepin-3-(benzo[b,e]oxepin-11(6H)-ylidene)-*N*,*N*-dimethylpropan-1-amine (DOX) is a traditional tricyclic antidepressant used to treat psychiatric syndromes over the past decades (5). It is commonly prescribed for the medication of depression and anxiety (6). It exhibits an effective central anticholinergic activity and has the ability to inhibit both norepinephrine and serotonin (5-HT) reuptake in synapses in brain (7). Generally, lower dosages of doxepin are recommended. Where the presenting symptoms are moderate in nature, it is advisable to start treatment at a dose of 10–50 mg daily. At high concentrations, serious adverse effects and toxicity can appear (8). Hence, the determination of doxepin is essential for obtaining optimum therapeutic concentration and for qualitative assurance in pharmaceutical preparations. Reported literature procedures generally focus on chromatographic techniques, including high-performance liquid chromatography (9-12), liquid chromatography-mass spectrometry (13), capillary gas-liquid chromatography (14), and extractive spectrophotometric methods (15). Chromatographic techniques are the most acceptable methods, where specificity is necessary. However, expensive instruments are needed and the experimental conditions are significantly affected by environmental factors. In addition, there are difficulties to detect some trialkylamines by use of UV absorption, because they do not adsorb very well in the UV–visible region, as a result of their low molar absorptivities (16). The chemiluminescence methods also commonly have relatively high detection limits (17). Since DOX is an electroactive molecule, electrochemical techniques provide a remarkable alternative approach to determine DOX due to their sensitivity, simplicity, fast response, and low costs. Nevertheless, according to our knowledge there are only a few literature reports studying the electrochemical properties of DOX. Among the few examples, polarography (18) ion-selective membrane electrode (19) and boron-doped diamond electrode (20) were all used to investigate DOX. However, their applications in monitoring of DOX might be limited due to toxicity of the dropping mercury electrode; sluggish response and inadequate detection limit (21). Thus, it is necessary to develop new determination procedures for DOX.

Carbon ionic liquid electrode (CILE) was proposed in 2006 for the first time as a new and high performance carbon composite electrode (22). The main idea for fabrication of this new electrode was the replacement of conventional nonconductive organic binders in carbon paste electrodes (CPEs) with a pyridinium-based ionic liquid. Some significant behaviors of CILE is wide potential window in aqueous solutions, renewable surface, low background current, resistivity toward bio-molecules fouling and a rapid electron transfer (23). Due to such prominent behavior CILEs can be used as appropriate sensors in electrochemical analysis of different biological compounds. 

In this manuscript we describe the application of CILE as an alternative appliance to study the electrochemical reaction of DOX as well as the application of differential pulse voltammetry (DPV) for sensitive determination of this biological material. The results suggest that the CILE can be effectively applied for both approaches including the study of electrochemical behavior and the up-growth of new analytical procedures to be applied for the determination of DOX.

## Experimental

*Reagents and Solutions*

Potassium dihydrogen phosphate, dipotassium hydrogen phosphate and graphite powder were purchased from Merck and were used as received. Doxepin was kindly supplied by Darou Pakhsh Pharmaceutical Company (Tehran, Iran) and used without prior purification. The ionic liquid, 1-octylpyridinum hexafluorophosphate, was synthesized as described elsewhere (24). Doxepin stock solution (0.01 M) was prepared with double distilled water and stored at 4 ºC in the dark. Phosphate buffer (PBS) 0.1 M, pH 8 was used as supporting electrolyte. All solutions were freshly prepared with double distilled water. The drug-free serum samples were kindly supplied by the Blood Transfusion Organization (Fars, Iran) and belonged to healthy male volunteers. The serum samples were stored frozen until the assay.

*Electrode Preparation*

CILE was fabricated by thoroughly hand-mixing the graphite powder and OPFP with a ratio of 50/50 (w/w) in a mortar and pestle, followed by packing the resulting paste firmly into the electrode cavity (1.8 mm ID) of a Teflon holder. In order to have better homogeneity in the composite and to lower background current, the electrode was heated for 2 min in oven, to a temperature above the melting point of IL (m.p. ~ 65 °C) prior to use (25). A copper wire inserted into the carbon paste provided the electrical contact. CPE was prepared by hand-mixing paraffin oil and graphite powder with 70/30 graphite/paraffin oil (w/w).

*Apparatus*

Voltammetric measurements were performed using an Autolab electrochemical system (Eco-Chemie, Utrecht, The Netherlands) equipped with Autolab PGStAT-302N, GPES software (Eco-Chemie, Utrecht, The Netherlands). The electrochemical cell was assembled with a conventional three electrode system: an Ag/AgCl/ KCl (3 M) reference electrode (Metrohm) and a platinum disk as a counter electrode. The working electrodes used in this study were GCE , CPE and CILE. All experiments were typically conducted at 25 °C without removing the dissolved oxygen. Scanning electron microscopy (SEM) images were obtained by using a HITACHI S-4160 field emission electronic microscopy (Japan). The FTIR studies were performed using a Perkin Elmer FT-IR spectrometer spectrum RX-1.

## Results and Discussion

*Surface Morphology and Structural Characterization of the Electrode*

**Figure 1:**
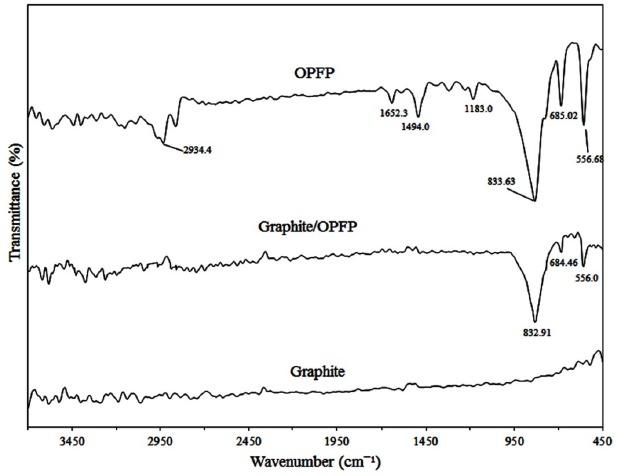
FTIR spectra of the graphite powder, graphite/OPFP and the OPFP respectively

**Figure 2:**
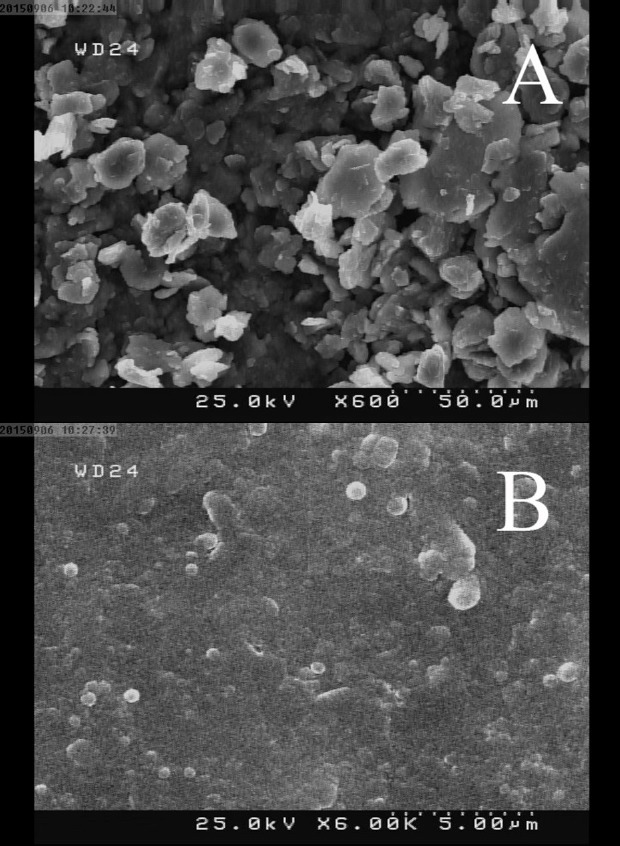
SEM images of (A) CPE and (B) CILE surfaces

To attain more insight about the structural characterization of the fabricated electrode, FTIR spectra of the graphite powder, OPFP and the graphite/OPFP were investigated and the results are illustrated in Figure 1. The peaks appeared at 2930 cm^-1^ (aliphatic C–H stretching), 1660 cm^-1^ (stretching vibration of C=N), 1490 cm^-1^ (in-plane bending vibration of –CH_3_, –CH_2_–) (26, 28) and 830 cm^-1^ (PF_6_¯) (27) which is in keeping with the literature data demonstrating the synthesis of the OPFP ionic liquid. Since graphite does not support a static dipole moment, the IR absorption peak of graphite is very weak (27). Therefore, the observed spectral features of the graphite/OPFP came from only the ionic liquid. The strong band at 833 cm^-1^ is assigned to the PF_6_¯ anion and can be seen in both OPFP and the graphite/OPFP spectra (27). The surface morphology of CILE and CPE was investigated by scanning electron microscopy. As may be seen in Figure 2 the electrode containing OPFP (B) as binder have clearly different morphology compared to the electrode used mineral oil as binder which has also been reported previously by other researchers (22, 25). The electrodes containing OPFP exhibited uniform surface morphology and unique structure, which reveal that the ionic liquid could fill well into the space between graphite particles.

*Electrocatalytic Properties of CILE toward Oxidation of *DOX

**Figure 3:**
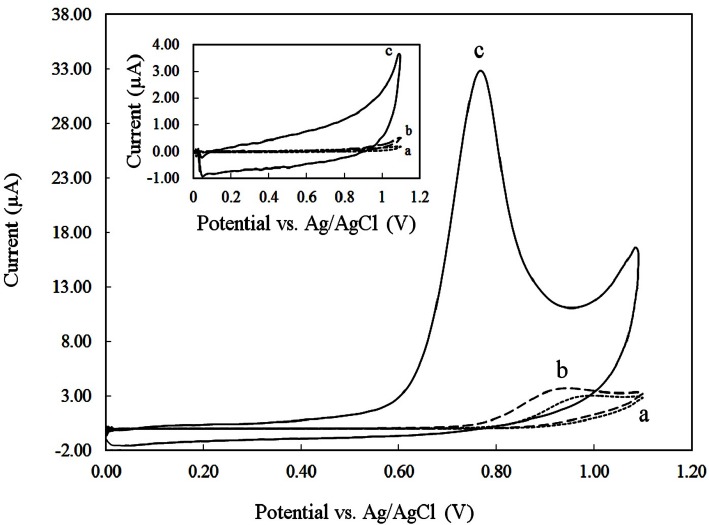
Cyclic voltammograms of different electrodes (a) CPE, (b) GCE, (c) CILE in PBS 0.1 M with pH 8 at a scan rate of 100 mVs-1; in the absence (inset) and presence of 250 µM DOX

Cyclic voltammograms were recorded at a potential sweep rate of 50 mVs^-1^ for a solution of 250 µM DOX in the 0.1 M phosphate buffer (pH 8) at CPE (Figure 3, curve a), GCE (Figure 3, curve b) and CILE (Figure 3, curve c). In the absence of DOX, no peak was observed on the CVs of the three electrodes (inset of Figure 3). However, in the presence of DOX an anodic peak was found. As seen a 10-fold increase in the oxidation peak current of DOX on CILE in comparison with CPE and GCE was occurred. Also the anodic peak potential of DOX on CILE (0.77V *vs.* Ag/AgCl) shifted towards more negetive potentials compared to the peak potentials of DOX on CPE (0.95 V) and GCE (0.93 V).

This effect is due to the electrocatalytic effect incited by the presence of ionic liquid used as a pasting binder instead of conventional mineral oils which present great electrocatalytic activities towards DOX oxidation. Furthermore, the ionic liquid used in the fabrication of the electrodes have properties like polar organic solvents. Therefore, DOX could be extracted from aqueous solution during adsorptive accumulation time and thus caused significant peak current enhancement at CILE. No cathodic peak of DOX was found on CPE, GCE, and CILE which suggests that the electrochemical oxidation of DOX is irreversible.

*Effect of the Solution pH*

**Figure 4:**
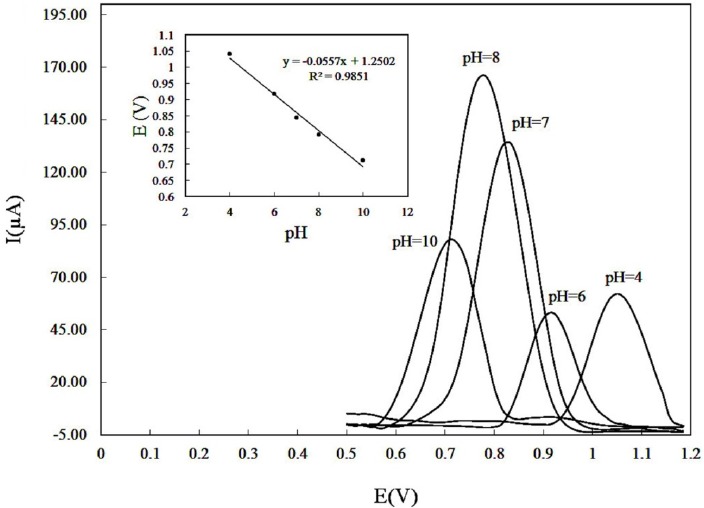
Effect of pH on the differential pulse voltammograms recorded for a DOX solution of 150 µM in PBS 0.1 M with accumulation potential of 0.3 V and accumulation time of 250 sec. pH values: 4.0, 6.0, 7.0, 8.0, 10. (Inset) plot of anodic peak potentials versus pH

As the electrode response of DOX can be affected by the solution pH, the influence of pH (4-10) on the anodic peak currents of solution containing 150 µM DOX was investigated (Figure 4). The maximum anodic current was obtained at pH 8.0. Therefore, pH 8 was selected as the optimum pH value. Moreover, by increasing pH the anodic peak potential shifted negatively (Figure 4 inset), following the linear equation: 

E_pa_ (V) = 1.2502 – 0.0557 pH; and R^2 ^= 0.9851.                                                 (1)

This suggests the presence of proton in the oxidation of DOX. Additionally, the slope of 0.0557 V/pH implies that equal number of electrons and protons involved in redox process according to the following equation (29):

0.0592 (h/n) V/pH                                                  (2)

where h and n are the number of protons and electrons involved in the electrode process, respectively. It looks acceptable that the mechanism of DOX oxidation includes the participation of one proton and one electron (16). Since the pK_a_ of 9.0 for DOX has been reported in literature (30), at much lower pHs, the oxidation of DOX normally became hard due to the strong protonation and in result the anodic responses were decreased. As the pH enhanced, the responses were increased due to the deprotonation (21). Neutral DOX is hydrophobic (21), and therefore at higher pH, the solubility diminished gently, which could decrease the responses of the electrode toward DOX.

*Sweep Rate Effect Studies*

**Figure 5:**
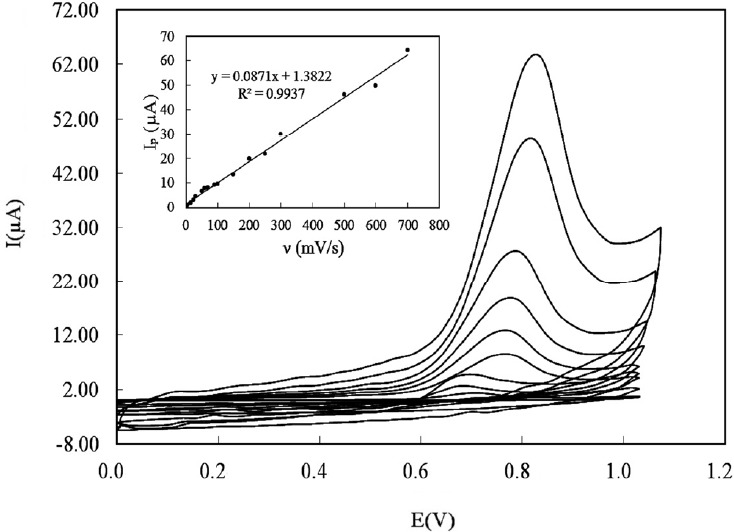
Cyclic voltammograms of 150 µM DOX in PBS 0.1 M with pH 8 using CILE recorded at various potential sweep rates over the range of (5-700 mVs-1). Inset: The plot of anodic peak current versus sweep rate

To study the predominant type of mass transport, the effect of sweep rates on the electrooxidation of DOX at the CILE was investigated using cyclic voltammetry over the range of 5-700 mVs^-1^ (Figure 5). The anodic peak of DOX grew by increasing the scan rate. It was found that the anodic peak current was linear to the scan rate in the mentioned range (Figure 5 inset) and the regression equation was:

i_p_ (µA) = 0.0871 ν (mVs^-1^) + 1.3822; and R^2 ^= 9937                                                  (3)

**Figure 6:**
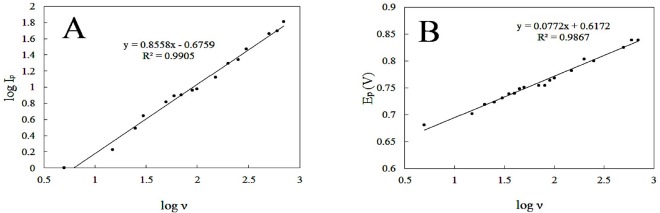
(A) linear dependence of log peak current to log scan rate in the range of 5-700 mVs-1 and (B) the plot of peak potential versus log sweep rate for 150 µM DOX in PBS 0.1 M with pH 8

This specifies the mass transport phenomenon in the oxidation process is adsorption-controlled. Moreover, Plot of log anodic peak current *vs.* log scan rate (Figure 6A) was also linear over the range of (5-700 mVs^-1^). For an ideal reaction of surface species a slope of 1.00 is expected and the slope of 0.86 is close to the expected value confirms that the DOX electrooxidation reaction is an adsorption controlled process.

According to Laviron’s theory (31), the relation between peak current and scan rate can be described as following equation: 

i_p _= n^2^F^2^vAΓ/4RT = nFQv/4RT                                                  (4)

**Scheme 1:**
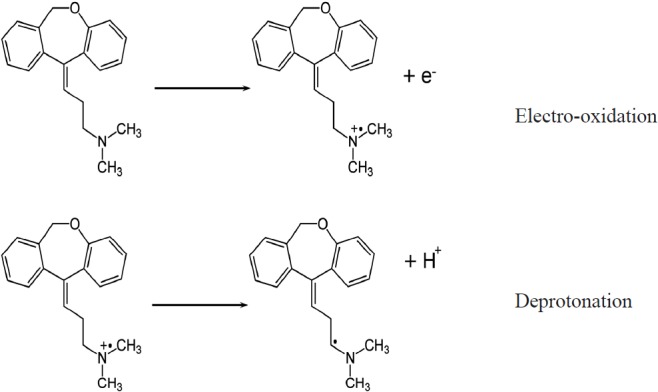
A proposed reaction mechanism for the electrooxidation of DOX.

Where A (= 0.02543 cm^2^) is the area of the electrode, n is the number of electrons, F is the Faraday constant, v is the scan rate, R is the gas constant, and T is the temperature. While the peak current (*i*_p_) and peak area (Q) are attained under specified scan rate (*v*) the number of electrons (n) can be calculated. Since the scan rates varied from 0.005 Vs^-1^ to 0.7 Vs^-1^, the average amount of n was calculated to be 0.97. This designates one electron is lost during the oxidation reaction. This result is in agreement with the DOX electrooxidation mechanism which has been reported in the literature (16). The electrooxidation of doxepin might take place at the nitrogen atom in the alkylamine, resulting in the formation of a cation radical, followed by deprotonation (Scheme 1). From the slope of the i_p_ versus *v *curve the surface concentration (Γ) of DOX was estimated about 2.3 × 10^-10^ mol cm^-2^.

Furthermore, by increasing scan rate, peak potential shifts to more positive values. This positive shift also demonstrates the irreversibility of the electrooxidation process. The anodic peak potential and the logarithm of scan rate also showed a linear relationship (Figure 6B), following the equation:

E_pa_ (V) = 0.0772 log*ν *+ 0.6172; and R^2 ^= 0.9867                                                   (5)

Tafel slope (b) can be obtained from the slope of anodic peak potential versus log of sweep rate using Equation 1 (32):

E_pa_= (b/2) log ν + ‏constant.                                                   (6)

Tafel slope was found to be 154 mV. The value of transfer coefficient (α) was calculated using the number of electrons that involved during DOX oxidation (n = 1) and the Tafel slope, according to following equation (33):

b = (2.303RT)/[(1-α)nF]                                                   (7)

the value of *α *= 0.62 was calculated.

Correspondingly, the following equation is driven from Laviron theory for an irreversible adsorption-controlled electrode reaction (31): 

Ep= E 0+2.203RT(1-α)nFlogRTks(1-α)nF+2.203RT(1-α)nFlogν

where I_p_, k_s_, and E^0’^ are the peak current, surface electron transfer rate constant, and formal potential, respectively. Meanwhile, by using the intercept of E_p_ versus log*ν* the value of k_s_ was determined to be 0.20 s^-1^. The E^0 ^= 0.75 V was determined from intercept of E_p_ *vs.* ν plot by extrapolating the line to be ν = 0.

*Influence of Accumulation Potential, and Accumulation Time*

The influence of accumulation potential and accumulation time on the oxidation peak current of DOX was studied. The accumulation potential was studied in the range of 0.1 to 0.7 V and highest anodic response was achieved for accumulation potential of 0.3 V. The accumulation time was also investigated in the range of 50 to 450 sec. The peak current was increased with increasing the accumulation time up to 250 sec and then decreased. This could be due to the adsorption of DOX and saturation of CILE surface. Additionally the optimal conditions for DPV response were recognized by measuring the current dependence on some instrumental parameters such as modulation amplitude, step potential, interval time and modulation time to obtain maximum signal to noise ratio and the optimum amounts were 70 mV, 10 mV, 0.1 sec and 0.05 sec, respectively.

*Differential Pulse Voltammetry of *DOX* at CILE*

**Figure 7:**
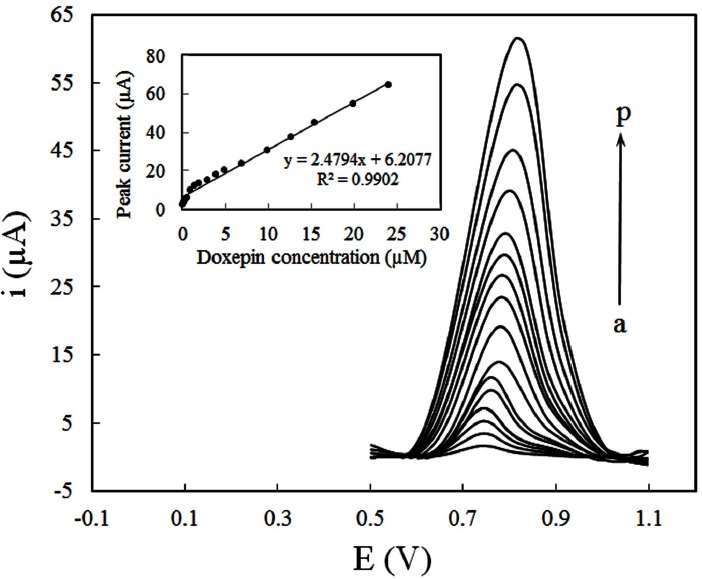
Differential pulse voltammograms for various concentrations of DOX (a) 0.048 (b) 0.12 (c) 0.24 (d) 0.48, (e) 0.95, (f) 1.44, (g) 1.92, (h) 2.87, (i) 3.9, (j) 5.14, (k) 6.87, (l) 9.84, (m) 12.59, (n) 15.4, (o) 19.9, (p) 24 µM, at CILE in 0.1M PBS (pH 8) accumulation potential of 0.3 V and accumulation time of 250 sec. Inset: Dependence of peak currents on the concentration of DOX

Under the selected analytical conditions, the quantification of DOX at different concentrations was performed by DPV. Figure 7 illustrates the differential pulse voltammograms recorded for a series of DOX solution with different concentrations in PBS (pH 8) and the respective calibration curve (inset). Good linearity was found in concentration range of 4.8 × 10^-8^ to 2.4 × 10^-5^ M. The linear regression equation was expressed as: 

i_p _(µA) = 2.4794 C_Doxepin_ (µM) + 6.2077 (R^2 ^= 0.9902).                                                   (9)

The detection limit (LOD) of the proposed method was estimated by the parameters obtained from the analytical curve, using LOD = 3S_b_/m, where S_b_ is the standard deviation of blank (n = 7) and *m* is the slope of calibration curve. Under the given conditions, the calculated LOD of DOX was found to be 2.1 × 10^−8^. The relative standard deviation (RSD) of the five times repeated measurement of 1 × 10^-5^ M DOX with the same electrode was 2.3%, whereas the RSD was 4.1% for 5 different electrodes. The analytical properties of current method are compared with the previous studies reported for the determination of DOX (Table 1). As mentioned before, the electrochemical quantification of DOX was seldom reported. Nevertheless, the CILE can easily be applied for this purpose with its better detention limit and wider linear range compared with previous works. 

The simple preparation and low cost material of the proposed electrode should also be considered.

**Table 1:** Comparison between different electrochemical methods used for the determination of DOX

**Method**	**Linear range (M)**	**Detection limit (M)**	**Reference**
CV using BDD electrode	5 × 10-8 to 1.5 × 10-4	9.2 × 10-8	(20)
Ion-Selective Membrane Electrode	1 × 10-2 to 4 × 10-5	1.5 × 10-5	(19)
Ad-DPV and Ad-SWV on GCE	5 × 10-6 to 1 × 10-5	2.4 × 10-7 and 1.5 × 10-7	(8)
DPV using sepeolite/CPE	3.6 × 10-7 to 4.2 × 10-6	1.4 × 10-7	(3)
DPV using CILE	5 × 10-8 to 2.4 × 10-5	2.1 × 10-8	This work

**Table 2:** Determination of DOX in pharmaceutical formulations

**No.**	**DOX added (µM)**	**DOX found ( µM)**	**Recovery (%)**
1	0	NDa	-
2	4.80	4.93 (±0.13)	102.7
3	9.60	9.47 (±0.15)	98.6
4	14.40	13.89 (±0.16)	96.45

a Not detected.

**Table 3:** Recovery study of DOX human blood serum sample

**No.**	**DOX added (µM)**	**DOX found** **a ** **( µM)**	**Recovery (%)**
1	0	4.83 (±0.14)	-
2	4.80	9.47 (±0.11)	96.7
3	9.60	14.76 (±0.13)	103.4
4	14.40	19.75 (±0.16)	103.61

a Average of three determination.

*Interference Studies*

To investigate the contaminant effect, the biological compounds normally existing in the serum samples including glucose, ascorbic acid, and uric acid were examined. Interference studies were conducted using a solution containing 10 µM DOX and interfering compounds. The tolerance limit was defined as the maximum concentration ratio of interfere/DOX caused an error less than *±*5.0% for the quantification of DOX. The results showed that 20-fold excess of glucose; 15-fold excess of ascorbic acid and 5-fold excess of uric acid did not interfere with the analysis of DOX.

*Real Sample Analysis*

To evaluate the applicability of the proposed method for the analysis of real samples, it was applied for DOX content in pharmaceutical preparations (nominal contain 4 mg DOX/tablet). Five tablets of DOX (Darou Pakhsh Pharmaceutical Company, Tehran, Iran) were accurately weighed and triturated to fine powder in a mortar. Then, precise amount of the powdered sample corresponding to a solution of 1 × 10^-4^ M DOX was dissolved in double distilled water by sonication for 10 min filtered into a 50 mL volume calibrated flask and diluted with double distilled water. A known volume of this solution was spiked into a 25 mL aliquot of the supporting electrolyte in the volumetric flask, followed by spikes of the standard DOX solution. 

The standard addition method was applied in these experiments. The amounts of DOX obtained in pharmaceutical formulations agree well with the label contents (Table 2). Additionally, the CILE was applied for the analysis of the human blood serum sample. A 10 mL human blood serum sample was deproteinized by adding 2 mL of 10% (w/w) trichloroacetic acid. Then, the solution was centrifuged and diluted 10 times with 0.1M PBS with pH 8.0. The appropriate amount of this diluted sample was transferred to the electrochemical cell for determination of DOX. The DPV measurements were done as described before. 

The results are presented in Table 3. The results presented good quantitative recoveries which indicate successful applicability of proposed method for real sample analysis.

## Conclusion

Carbon ionic liquid electrode can be applied as an analytical sensor for electrochemical determination of DOX. The electrode revealed excellent response compared with conventional electrodes. We have also presented an appropriate analytical methodology for highly sensitive quantitative determination of DOX, based on differential pulse voltammetry which showed the preeminent performance due to electrocatalytic effect of the proposed electrode.
